# The enhancement of nodal properties in the dorsal visual pathway is associated with compensatory mechanisms of visuospatial cognitive abilities following total sleep deprivation

**DOI:** 10.3389/fnins.2025.1585763

**Published:** 2025-09-04

**Authors:** Zhenyu Han, Zihan Gang, Mengke Ma, Yongcong Shao

**Affiliations:** ^1^College of Traditional Chinese Medicine, Liaoning University of Traditional Chinese Medicine, Shenyang, China; ^2^School of Psychology, Beijing Sport University, Beijing, China; ^3^Key Laboratory for Biomechanics and Mechanobiology, Ministry of Education, School of Biological Science and Medical Engineering, Beihang University, Beijing, China

**Keywords:** dorsal visual pathway, total sleep deprivation, resting-state electroencephalogram, mental rotation, graph theory

## Abstract

**Introduction:**

Vision is the most critical sensory perception in humans, however, total sleep deprivation (TSD) impairs visuospatial cognitive abilities. While evidence suggests that the brain actively counteracts the adverse effects of TSD to preserve visual cognition, the underlying neural mechanisms remain poorly understood.

**Methods:**

To explore the compensatory mechanisms of TSD on visuospatial cognition, we collected resting-state electroencephalogram (EEG) data from 24 participants at baseline state (BS) and after 36 h of TSD, along with behavioral data from a mental rotation task. Graph theory-based analyses were employed to assess global and nodal network properties.

**Results:**

Behaviorally, reaction time (RT) significantly increased under TSD compared to BS, indicating impaired spatial cognition. In contrast, accuracy (ACC) for mirrored 120° trials significantly improved under TSD, suggesting that participants invested greater effort in more demanding tasks and adopted a strategy of prolonged RT to maintain ACC. EEG findings revealed a significant increase in the small-worldness index (sigma) after TSD, which positively correlated with the improvement in ACC for mirrored 120° trials. Nodal properties in the dorsal visual pathway, particularly in parietal regions, were significantly enhanced following TSD. Similarly, nodal properties in the left middle frontal gyrus and left superior temporal gyrus were significantly strengthened, and these enhancements were positively associated with the increase of ACC in mirrored 120° trials.

**Discussion:**

These results demonstrate that individuals compensate for TSD-induced visual cognitive deficits by enhancing small-world network property and information transfer efficiency in the dorsal visual pathway. Additionally, top-down control is mediated by the middle frontal gyrus, while bottom-up information integration is facilitated by the superior temporal gyrus.

## Introduction

Visual function is a critical capability for survival, playing an irreplaceable role in gathering environmental information and discerning danger signals. This necessitates that, even under extreme conditions such as cognitive resource depletion, extreme fatigue, or impaired consciousness, the organism must still allocate precious and limited resources to maintain visual function (Schmidt, 2014; Cai et al., 2021).

The visual pathway is divided into two distinct streams: the dorsal visual pathway, often referred to as the “where” pathway, and the ventral visual pathway, known as the “what” pathway (Ungerleider and Mishkin, 1982). The dorsal pathway includes several key regions: the primary visual cortex V1 (Broadman Area 17), the secondary visual cortex V2 (Broadman Area 18), the middle temporal area, the superior medial temporal lobe (Nassi and Callaway, 2009; Rizzolatti and Matelli, 2003), the superior parietal lobule, and the inferior parietal lobule (Randerath et al., 2010; Salazar-López et al., 2016). In contrast, the ventral pathway originates from V1, progresses through V2, and ultimately reaches the inferior temporal lobe (Kaas and Lyon, 2007). Research indicates that the dorsal pathway facilitates the holistic processing of visual stimuli, while object recognition and the processing of local visual information are primarily managed by the ventral pathway (Collins et al., 2019; Serre et al., 2005). Additionally, the dorsal visual pathway is involved in processing visual motion information, spatial localization, and selective visual attention (Goodale and Westwood, 2004).

Quality sleep is fundamental to maintaining both physical and mental health. However, factors such as irregular sleep schedules, prolonged work-related stress, and the use of electronic devices have led to an increasingly prevalent issue of TSD. TSD has been shown to significantly impair alertness and attention (Philibert, 2005; Lim and Dinges, 2010), which are basic components of higher cognitive functions. This impairment can lead to widespread cognitive deficits, affecting abilities such as emotional regulation (Tempesta et al., 2010), decision-making (Harrison and Horne, 2000; Whitney et al., 2015), and working memory (Tucker et al., 2010).

Furthermore, numerous studies have demonstrated that TSD impairs visual cognition, manifesting as diminished performance in visual working memory (Lim et al., 2007, 2010), visual attention (Zeng et al., 2021), and other visually-dependent cognitive tasks (Asplund and Chee, 2013; Chee et al., 2011). Additionally, both resting-state and task-based studies have revealed significant reductions in activation within the visual cortex (Vuilleumier and Pourtois, 2007; Fusar-Poli et al., 2009), the ventral visual pathway (Lim et al., 2010; Van Dongen et al., 2011), and the dorsal parietal regions (Chuah and Chee, 2008) following TSD. The detrimental effects of TSD on visual cognition can be attributed to the depletion of neural resources (Shaw et al., 2009). Cognitive tasks require sustained activation of visual attention circuits, leading to reduced sensitivity of the visual cortex to sensory stimuli (Chee et al., 2008) and increased fatigue (Van Dongen et al., 2011). Some researchers argue that this impairment in visual cognition is due to a decline in top-down attentional control within the visual pathways, as multiple studies have reported decreased activation in the frontoparietal attention network following TSD (Tomasi et al., 2009; Kong et al., 2012).

However, studies investigating changes in visual cortex activation following TSD have yielded inconsistent results. Some researchers have found that only extrastriate visual regions exhibit reduced activation after TSD, while early visual cortex activity remains unchanged (Chee and Tan, 2010; Chee et al., 2011). In contrast, other fMRI studies report no significant changes in visual cortex activation post-TSD (Poudel et al., 2012) or even increased regional spontaneous neural activity and short-term functional connectivity (FC) (Kong et al., 2018; Dai et al., 2012). These discrepancies may stem from methodological differences across studies or could reflect compensatory mechanisms employed by the brain to counteract the cognitive impairments caused by TSD.

Numerous studies have provided substantial evidence supporting this point. In healthy individuals, while performance on visual attention tasks deteriorates following TSD, feature-based visual search remains relatively preserved (Horowitz et al., 2003). Effective cues can enhance task performance, and this cueing effect persists even after TSD (Versace et al., 2006), accompanied by regulatory activation in the parahippocampal gyrus (Lim et al., 2010). In patients with chronic insomnia, hyperactivation in the visual cortex has been observed (Dai et al., 2016), which is considered a contributing factor to sleep initiation difficulties (O’Byrne et al., 2014). Furthermore, in patients with visual cortex damage, unconscious visual experiences can still facilitate facial emotion recognition and activate subcortical pathways (Motomura et al., 2014). These consistent findings across different populations demonstrate the prevalence of compensatory responses to visual cognitive impairments. Despite facing cognitive resource limitations following TSD, individuals consistently demonstrate the capacity to maintain visual cognitive functions through various compensatory mechanisms.

The mental rotation task is a widely used paradigm for assessing visual-spatial cognitive abilities. Neuroimaging studies have shown that this task robustly activates both the dorsal and ventral visual pathways. The dorsal pathway is primarily responsible for processing spatial information, whereas the ventral pathway plays a key role in object recognition (Booth et al., 2000; Koshino et al., 2005). Furthermore, the prefrontal cortex is strongly implicated in the mental rotation task (Ecker et al., 2008), contributing to the comparison of angular differences between objects before and after rotation, as well as managing spatial working memory (Gogos et al., 2010) and attentional control (Koshino et al., 2005). The parietal cortex, on the other hand, is primarily involved in generating visual rotations (Podzebenko et al., 2005) and integrating visual information (Gogos et al., 2010).

Previous literature has indicated that there is indeed a certain compensatory effect in the visual cortex after TSD, but the specific brain regions, frequency bands, and compensation methods involved remain unknown. To investigate the neural mechanisms underlying the impairment of visual-spatial cognitive abilities due to TSD, we utilized a mental rotation task to assess the effects of TSD on behavioral performance. Additionally, we applied graph theory methods to analyze differences in activity within the visual dorsal and ventral pathways during both BS and 36-h TSD states. Our aim was to identify potential compensatory mechanisms employed by the brain to mitigate the impact of TSD on visual-spatial cognition. This study was guided by the following hypotheses: (1) TSD would result in poorer performance on the mental rotation task, specifically manifesting as reduced ACC and prolonged RT; (2) Based on previous fMRI studies (Lim et al., 2010; Asplund and Chee, 2013), we hypothesized that TSD would lead to decreased graph theory metrics (including sigma, degree centrality, nodal efficiency, etc.) in the parietal and frontal regions associated with mental rotation; (3) both the ventral and dorsal visual pathways would exhibit compensatory responses following TSD, for instance, some graph theory metrics (including sigma, degree centrality, nodal efficiency, etc.) would be enhanced after TSD.

## Materials and methods

### Participants

Based on the G*Power 3.1.9.7 software, we calculated that at least 24 participants were required for this experiment. Therefore, we recruited a total of 24 healthy adults (12 women, 12 men; mean age = 22.71 years). Over the 36-h TSD period, participants completed two mental rotation tasks and underwent two sessions of resting-state EEG data collection. Due to excessive noise in the EEG recordings, data from 3 participants were excluded, leaving 21 participants for the EEG data analysis. Additionally, because of missing behavioral data, 23 participants were ultimately included in the behavioral analysis.

Participants were excluded from recruitment based on the following criteria: (1) non-right-handedness; (2) Abnormal vision or corrected vision; (3) age outside the range of 20–28 years; (4) a score of ≥5 on the Pittsburgh Sleep Quality Index assessed prior to the experiment; (5) a history of mental illness; (6) a history of sleep-related disorders or significant physical illnesses; (7) use of sleep or psychiatric medications within 1 month before the experiment; (8) regular consumption of coffee, tea, or alcoholic beverages.

The study protocol was approved by the Biological and Medical Ethics Committee of Beihang University (ethics code: BM20180040). All participants provided written informed consent prior to the experiment and received monetary compensation upon completion of the study.

### Experiment implementation

**FIGURE 1:**
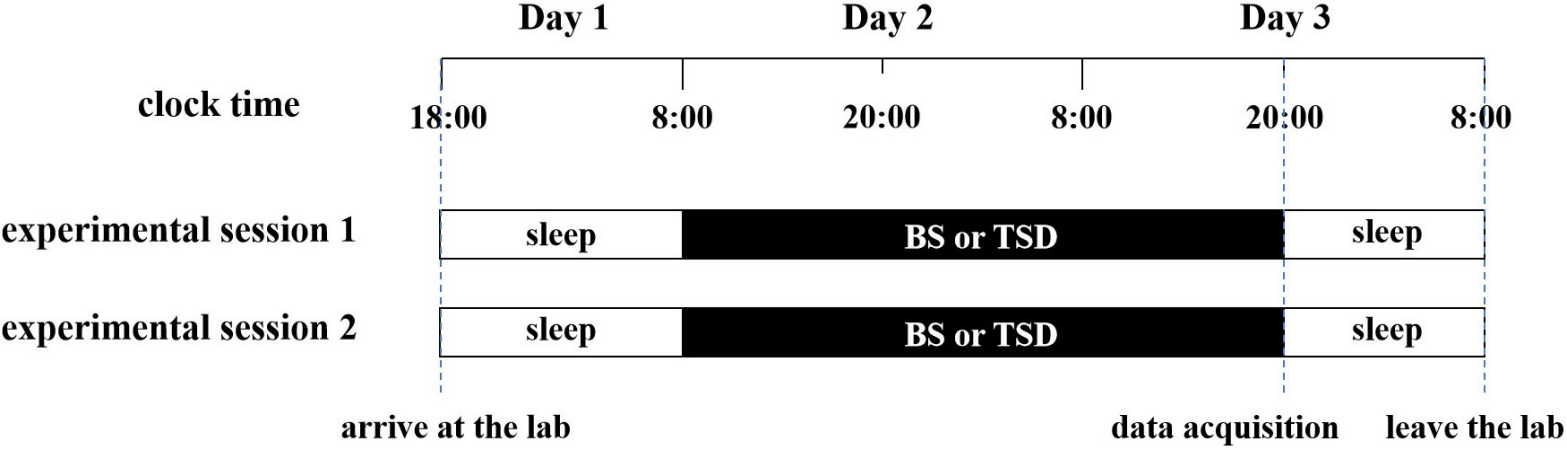
Total sleep deprivation (TSD) experiment flow chart.

Twenty-four participants were randomly allocated to two experimental groups. Group 1 participated in data collection under the BS condition initially, followed by the TSD condition after a 1-month interval. Group 2 followed the inverse order. To avoid the impact of sudden arrival in the new environment on individuals and improve the familiarity of participants with the experimental environment and experimental procedures, participants were required to arrive at the laboratory at 18:00 on the first day (Figure 1). The experimenters explained the experimental procedures and important considerations to the participants, who then signed the informed consent forms and spent the night in the laboratory. The TSD experiment officially began at 8:00 AM on the second morning and concluded at 8:00 PM on the third evening. All data collection was conducted at 8:00 PM on the third day. To prevent the mental rotation task from affecting the resting state, all participants collected resting-state data prior to the completion of behavioral tasks. During the experiment, if the participants were found to show signs of sleepiness, such as closing their eyes, nodding their heads, etc., the researchers would promptly guide them to simple physical activities (such as walking, doing simple stretching exercises) through gentle reminders to keep them awake.

During the 4-weeks interval between experimental sessions, participants were instructed to adhere to specific guidelines: no consumption of medications, alcohol, coffee, tea, milk tea, or other caffeinated beverages; no participation in strenuous physical activity; and strict maintenance of a regular sleep schedule. Researchers monitored compliance by requiring participants to complete daily sleep logs. These logs confirmed that all participants retired to bed by 23:00 each evening and achieved a minimum of 7.5 h of sleep per night throughout the study period.

### EEG recording

Participants completed a 10-min session of resting-state EEG data collection, during which they were instructed to close their eyes, avoid frequent eye movements, and minimize physical activity and muscle tension. Subsequently, they performed the mental rotation task.

Data acquisition was conducted using a Scan 4.5 64-electrode EEG system, which complies with the international 10–20 electrode system standards. The sampling rate was set at 1000 Hz, with reference electrodes placed on both mastoids. Two horizontal electrooculogram (EOG) electrodes were placed two fingers below the eyebrows, approximately 1 cm from the outer corner of the eye. Two vertical EOG electrodes were positioned above the eyebrow and below the lower eyelid, respectively, to record eye movements. Before placing the bilateral mastoid electrodes and EOG electrodes, an abrasive paste was applied to exfoliate the skin and remove dead cells.

### Mental rotation task

The stimulus images consisted of two letter “R”s and a “+” sign. The “+” was displayed at the center of the screen, with a letter “R” rotated 150° counterclockwise on the left side, which remained constant across all trials. On the right side, the letter “R” was presented in one of eight possible orientations: it was either rotated clockwise by 0°, 60°, 120°, or 180° relative to the left image, or it was first mirrored and then rotated clockwise by the same angles. The mental rotation task required participants to determine whether the right-side letter “R” was a mirrored version of the left-side image. If it was not mirrored, participants were instructed to press the “F” key; if it was mirrored, they were to press the “J” key.

There were a total of 2 (Normal, Mirrored) × 4 (0°, 60°, 120°, 180°) types of stimulus images, each randomly presented 32 times, resulting in 256 trials. In each trial, a “+” sign was first displayed at the center of the screen for a randomly determined duration between 200 and 500 ms. This was followed by the presentation of the stimulus image, during which participants were required to respond as quickly and accurately as possible by pressing the appropriate key. The stimulus would disappear automatically after a key press or if no response was made within 2000 ms. Prior to the formal experiment, a practice session was conducted to familiarize participants with the task rules.

### EEG data analysis

We preprocessed the EEG data using the EEGLAB toolbox in MATLAB (2013b). The raw data were first subjected to a bandpass filter of 0.1–40 Hz and a notch filter of 48–52 Hz to remove line noise. The data were then segmented into 2000 ms epochs. Following this, each epoch was visually inspected: channels exhibiting significant signal drift were interpolated, with an average of 2.82 components interpolated per participants, and epochs containing obvious artifacts were removed. Independent component analysis (ICA) was subsequently performed to identify and eliminate artifact components. We deleted the obvious artifact components, including six classic types of artifact components such as blinks, eye drifts, power frequency interference, electromyography, electrocardiogram, and poor electrode contact. For the components that could not be identified or were uncertain whether they were artifacts, we all retained them. On average, 3.73 components were deleted per subject. Epochs with voltage values outside the range of −100 to 100 μV were excluded, and the data were re-referenced to the average of all channels. Finally, the sampling rate was downsampled to 250 Hz, resulting in clean EEG data for further analysis.

We utilized the FieldTrip toolbox to compute FC matrices. First, the “mtmfft” method in the “ft_freqanalysis” function was employed to perform a fast Fourier transform on the data across four frequency bands: delta (1 Hz ≤ delta < 4 Hz), theta (4 Hz ≤ theta < 8 Hz), alpha (8 Hz ≤ alpha < 12 Hz), and beta (12 Hz ≤ beta < 30 Hz). Next, the forward solution was calculated using the “ft_prepare_leadfield” function, followed by source analysis using the “ft_sourceanalysis” function. FC was then computed based on the Anatomical Automatic Labeling 90 (AAL-90) atlas and the phase-locking value (PLV) method, resulting in a 90 × 90 FC matrix for each participant. We used the AAL-90 atlas because it is the most widely used atlas in cognitive neuroscience research. Moreover, it has fewer brain regions, which can reduce the complexity of calculations and the precision required for computer configuration. We chose the PLV index because, compared with other FC indicators, it is completely insensitive to amplitude fluctuations, can detect both linear and non-linear phase synchronization, can directly and unbiasedly measure the phase locking strength, and its calculation is relatively simple and efficient.

Subsequently, graph theory analysis was performed on the FC matrices using the GRETNA toolbox. Before introducing the specific calculation process, we needed to first explain a few concepts. Path length refers to the number of edges connecting two nodes, with the shortest path length of a node representing the minimal number of edges required to connect it to all other nodes. The clustering coefficient of a node reflects the likelihood that neighboring nodes are connected to it. A small-world network is a unique network structure that exhibits higher information transmission efficiency compared to random or regular networks, quantified by the sigma value. σ = γ/λ, where λ is the ratio of the shortest path length of the real network to that of the random network; γ is the ratio of the clustering coefficient of the real network to that of the random network. Since small-world networks have a high clustering coefficient and a shorter shortest path length, while the shortest path length of the random network is similar to that of the small-world network, the clustering coefficient of the random network is much smaller than that of the small-world network, so γ is much greater than 1 and λ ≈ 1. Thus, the small-world network index σ being greater than 1 conforms to the properties of the small-world network. To eliminate the influence of random fluctuations, the σ threshold is finally defined as 1.1.

In addition, Network Efficiency, which includes global and local efficiency, measures the network’s capacity for information transfer. Betweenness Centrality quantifies the frequency at which a node appears on the shortest paths between other nodes. The degree centrality of a node refers to the number of edges connected to it by all nodes in the network. Nodal efficiency decreases as the average path length between a node and others increases, indicating weaker information transmission capacity. Finally, nodal local efficiency represents the efficiency of connections among a node’s neighboring nodes.

When calculating the above graph theory indicators, the small-world property was first evaluated across all participants using a sparsity range of 0.0507:0.05:0.5, yielding sigma values. Then the maximum sparsity range was selected under the condition that all participants’ sigma values exceeded 1.1, ensuring the presence of small-world properties. Based on the chosen sparsity range, global properties (Small World, Network Efficiency) and nodal properties (Betweenness Centrality, Degree Centrality, Nodal Cluster Coefficient, Nodal Efficiency, and Nodal Local Efficiency) were calculated.

### Statistical analysis

Behavioral data were statistically analyzed using SPSS 27. A repeated-measures analysis of variance (ANOVA) was conducted for both RT and ACC in the mental rotation task, with a 2 (BS, TSD) × 2 (Normal, Mirrored) × 4 (0°, 60°, 120°, 180°) design. *Post hoc* comparisons were corrected using the Holm method for multiple comparisons.

For graph theory metrics, global and nodal properties were analyzed using the Metric Comparison module in GRETNA. Paired *t*-tests were applied, with no multiple comparison correction for global properties, while nodal properties were corrected using the false discovery rate (FDR) method.

Pearson correlation analysis was conducted to assess the association of graph theory metric differences and behavioral differences between BS and TSD. Multiple comparisons were corrected using the FDR method. The significance level was set at 0.05 for all analyses.

## Results

### Behavioral results

Descriptive statistical results for RT and ACC are presented in Tables 1, 2. A repeated-measures ANOVA with a 2 (BS, TSD) × 2 (Normal, Mirrored) × 4 (Angle: 0°, 60°, 120°, 180°) design was conducted for both RT and ACC.

**TABLE 1:** RT results of mental rotation task.

	RT (ms)			
Angle	Normal		Mirrored	
	0 h	36 h	0 h	36 h
0°	585.468 ± 94.883	611.642 ± 139.475	663.925 ± 91.579	709.497 ± 209.284
60°	772.361 ± 123.877	876.955 ± 219.877	870.420 ± 149.746	1038.849 ± 307.662
120°	874.194 ± 172.222	1068.481 ± 293.251	904.329 ± 185.832	1092.010 ± 320.865
180°	668.048 ± 91.986	718.360 ± 170.796	742.516 ± 94.950	747.092 ± 186.181

**TABLE 2:** ACC results of mental rotation task.

	ACC			
Angle	Normal		Mirrored	
	0 h	36 h	0 h	36 h
0°	0.978 ± 0.024	0.973 ± 0.039	0.907 ± 0.206	0.932 ± 0.206
60°	0.814 ± 0.203	0.799 ± 0.221	0.693 ± 0.245	0.752 ± 0.238
120°	0.61 ± 0.204	0.537 ± 0.228	0.631 ± 0.252	0.667 ± 0.278
180°	0.929 ± 0.056	0.923 ± 0.079	0.882 ± 0.201	0.891 ± 0.213

**FIGURE 2:**
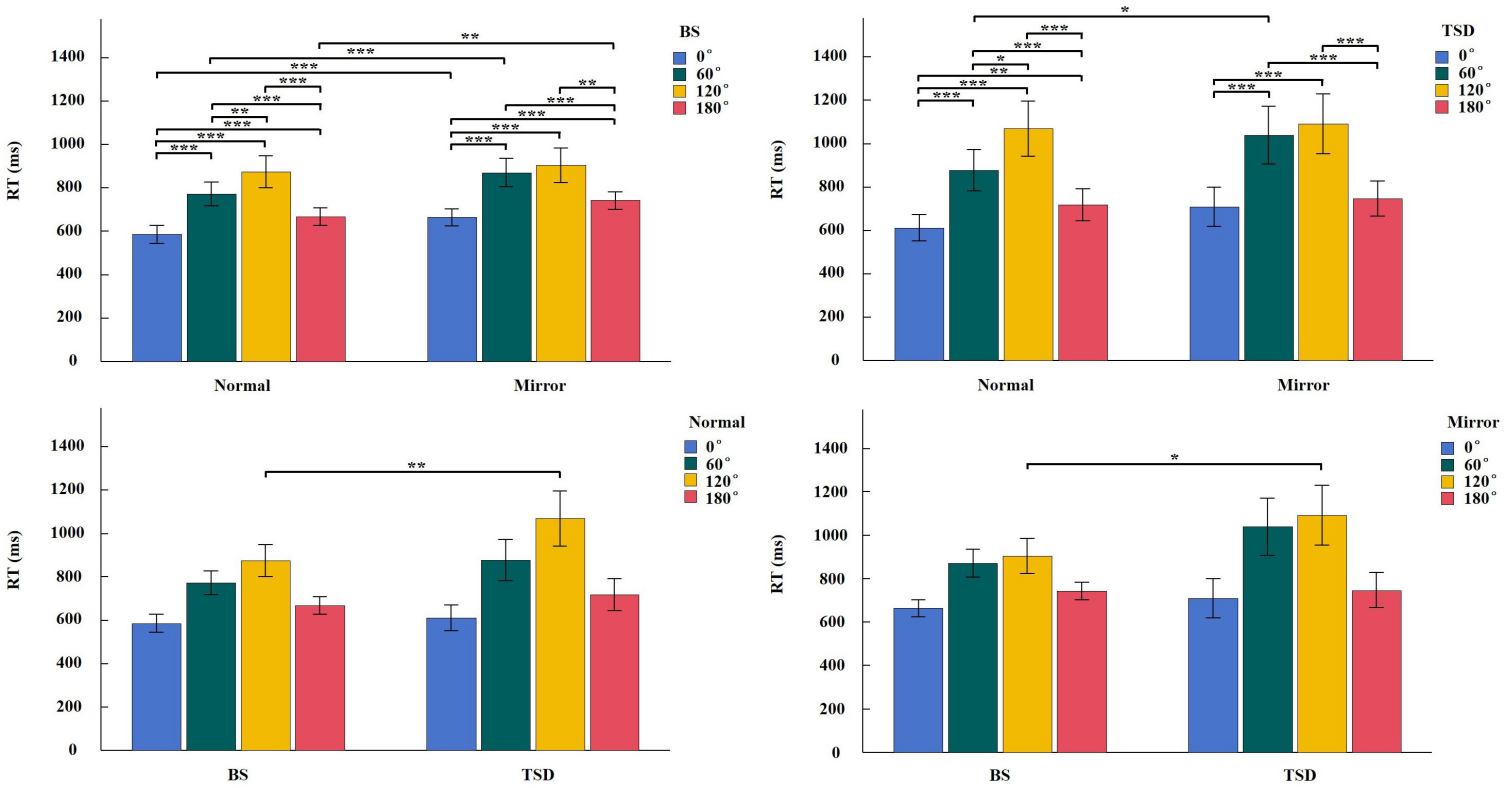
Simple effect analysis of RT interaction in Sleep condition × Image type × Angle. *p < 0.05, **p < 0.01, ***p < 0.001.

For RT, the analysis revealed a significant main effect of sleep condition, *F*_(1_,_22)_ = 9.383, *p* = 0.006, η^2^ = 0.060, with significantly longer RT under TSD compared to BS. A significant main effect of image type was also observed, *F*_(1_,_22)_ = 22.201, *p* < 0.001, η^2^ = 0.034, with longer RT for mirrored images compared to normal images. Additionally, a significant main effect of angle was found, *F*_(3_,_66)_ = 77.053, *p* < 0.001, η^2^ = 0.459. *Post hoc* comparisons indicated the following order of RT: RT(120°) > RT(60°) > RT(180°) > RT(0°). The interaction between sleep condition and image type was not significant, *F*_(1_,_22)_ = 0.098, *p* = 0.758, η^2^ < 0.001. However, significant interactions were observed between sleep condition and angle (Supplementary Figure 1), *F*_(3_,_66)_ = 14.426, *p* < 0.001, η^2^ = 0.030; between image type and angle (Supplementary Figure 2), *F*_(3_,_66)_ = 11.714, *p* < 0.001, η^2^ = 0.009; and among sleep condition, image type, and angle (Figure 2), *F*_(3_,_66)_ = 3.546, *p* = 0.019, η^2^ = 0.002.

**FIGURE 3:**
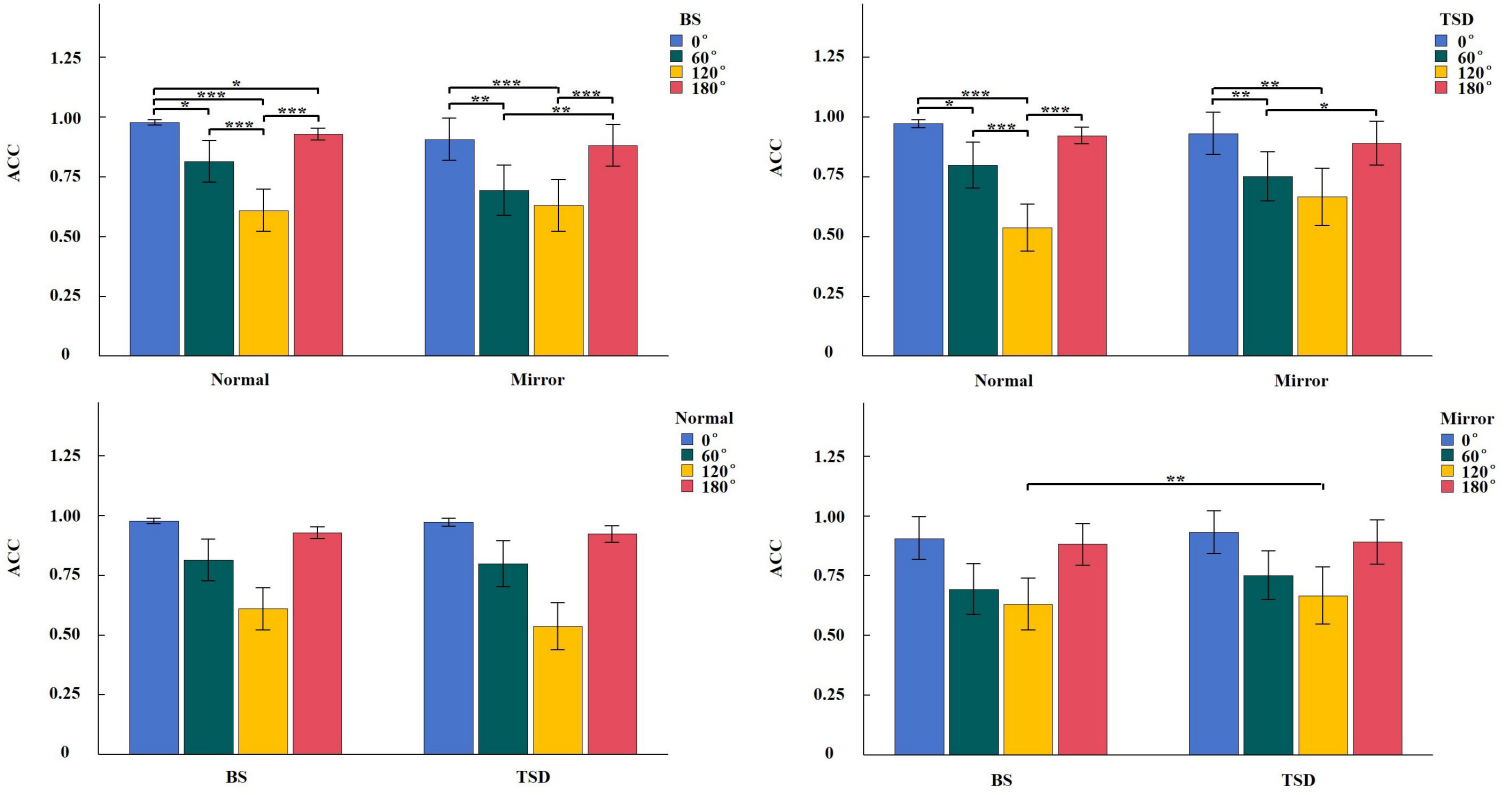
Simple effect analysis of ACC interaction in Sleep condition × Image type × Angle. *p < 0.05, **p < 0.01, ***p < 0.001.

For ACC, the main effect of sleep condition was not significant, *F*_(1_,_22)_ = 0.218, *p* = 0.645, η^2^ < 0.001. Similarly, the main effect of image type was not significant, *F*_(1_,_22)_ = 0.242, *p* = 0.628, η^2^ = 0.004. However, a significant main effect of angle was observed, *F*_(3_,_66)_ = 100.922, *p* < 0.001, η^2^ = 0.381. *Post hoc* comparisons revealed the following order of ACC: ACC(120°) < ACC(60°) < ACC(180°) < ACC(0°). Significant interactions were found between sleep condition and image type (Supplementary Figure 3), *F*_(1_,_22)_ = 27.732, *p* < 0.001, η^2^ = 0.004; between image type and angle (Supplementary Figure 4), *F*_(3_,_66)_ = 3.681, *p* = 0.016, η^2^ = 0.020; and among sleep condition, image type, and angle (Figure 3), *F*_(3_,_66)_ = 3.634, *p* = 0.017, η^2^ = 0.002; while the interaction between sleep condition and angle was not significant, *F*_(3_,_66)_ = 1.131, *p* = 0.343, η^2^ = 0.001.

### EEG results

A paired-sample *t*-test was conducted to compare global and nodal properties between the BS and TSD states across four frequency bands. For nodal properties, FDR correction was applied for multiple comparisons. The analysis of global properties revealed that the small-world index, sigma, showed significant differences in the delta, theta, and beta frequency bands, with sigma values under TSD being significantly higher than those under BS. Specifically, in the delta band, *t* = −3.475, *p* = 0.002; in the theta band, *t* = −4.725, *p* < 0.001; and in the beta band, *t* = −3.126, *p* = 0.006.

**FIGURE 4:**
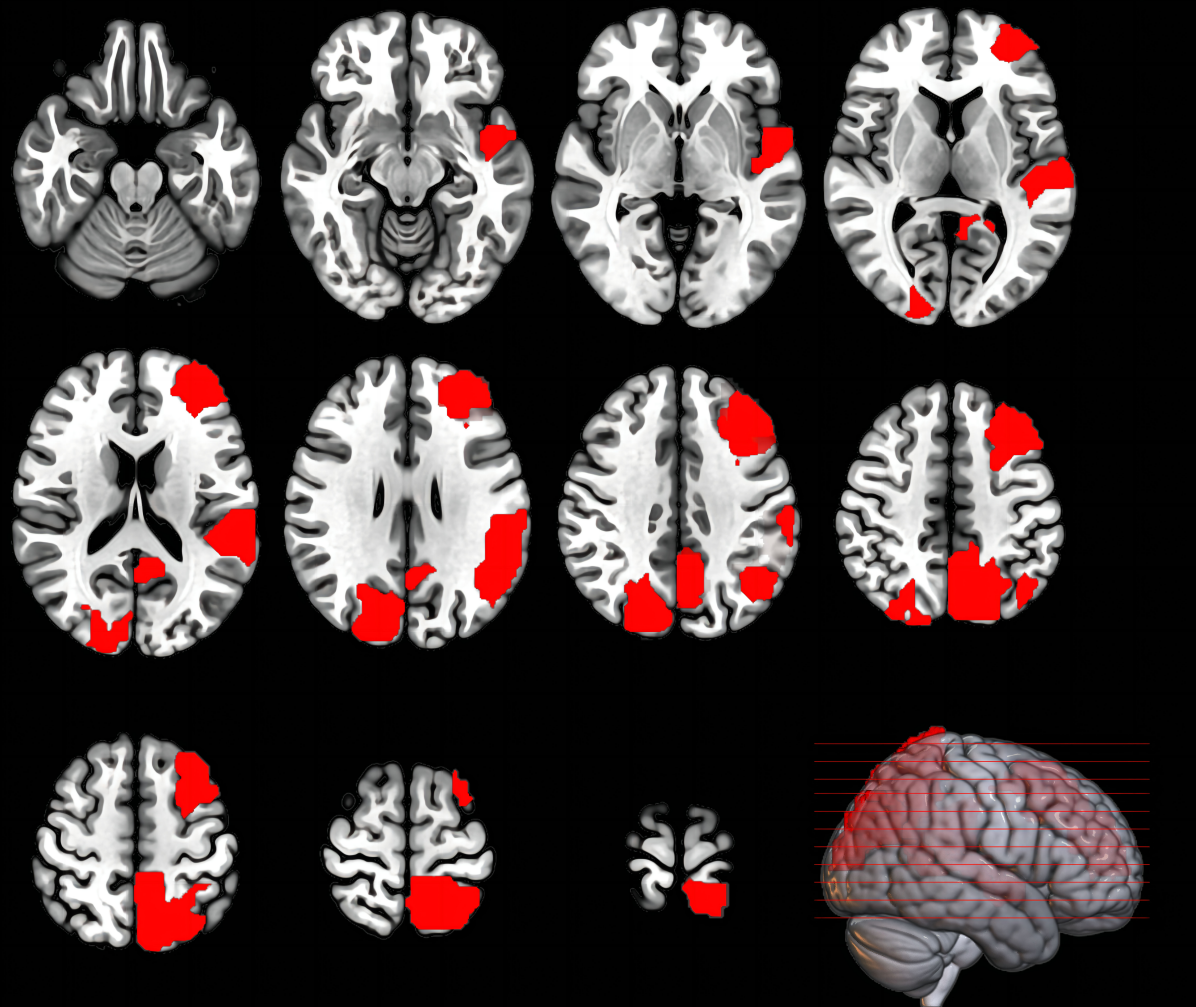
Brain regions where the nodal properties were significantly enhanced in the TSD state compared to the BS state. This image shows the axial plane of the brain, with the left hemisphere on the right.

Regarding nodal properties (Figure 4 and Table 3), the results mainly highlighted cases where the *t*-values were negative (indicating BS < TSD). The complete results can be found in the Supplementary material (Supplementary Tables 1–4).

**TABLE 3:** Paired *t*-test statistical results for nodal properties with negative *t*-values.

Frequency band	Brain regions	p(Dc)	t(Dc)	p(Ne)	t(Ne)	p(NLe)	t(NLe)
Delta	SupraMarginal_L	<0.001	−4.245	<0.001	−4.094	\	\
	Angular_L	0.005	−3.123	\	\	\	\
Theta	Parietal_Sup_L	\	\	0.008	−2.961	\	\
	SupraMarginal_L	\	\	0.006	−3.085	\	\
	Angular_L	\	\	0.009	−2.911	\	\
	Precuneus_L	0.004	−3.228	0.008	−2.944	\	\
Alpha	Frontal_Mid_L	\	\	0.002	−3.649	\	\
	SupraMarginal_L	\	\	0.001	−4.080	\	\
	Precuneus_L	\	\	0.005	−3.187	\	\
	Temporal_Sup_L	\	\	\	\	0.003	−3.345
Beta	Cuneus_R	0.004	−3.303	\	\	\	\
	Occipital_Sup_R	0.005	−3.237	0.004	−3.292	\	\
	SupraMarginal_L	0.002	−3.523	\	\	\	\

Dc, degree centrality; Ne, nodal efficiency; NLe, nodal local efficiency; “\”means no significant results.

### Correlation results

Nodes with negative *t*-values were selected from the nodal properties, and their differences (TSD - BS) were calculated. These selected nodal differences, along with the differences in sigma values, were then calculated the correlation with the differences (TSD - BS) in RT for both normal and mirrored 120° trials, as well as ACC in the mirrored 120° trials. FDR correction was applied for multiple comparisons.

**FIGURE 5:**
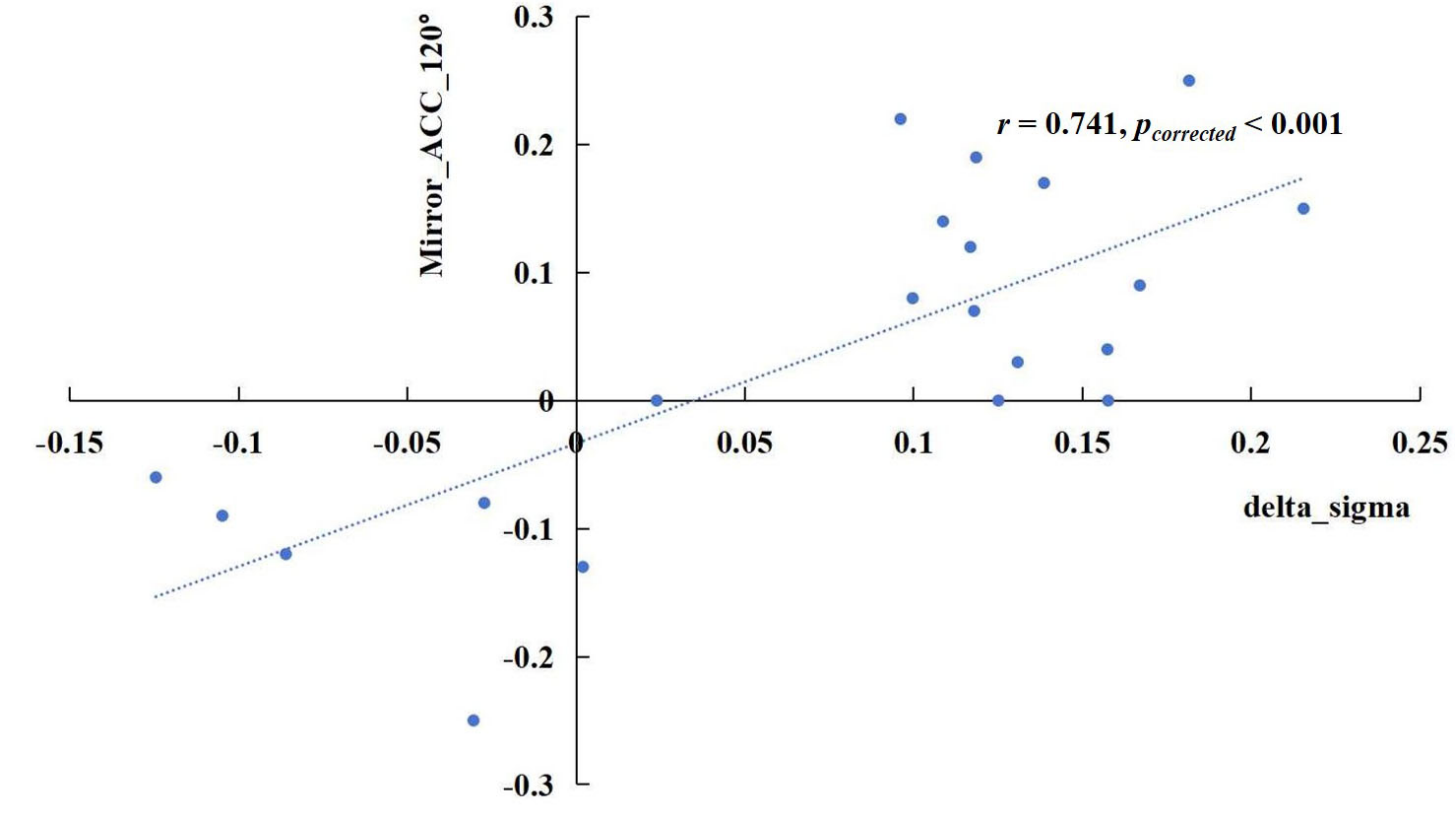
The difference of sigma value in the delta band between the BS and TSD state was positively correlated with the difference of ACC in the mirrored 120° trials.

**FIGURE 6:**
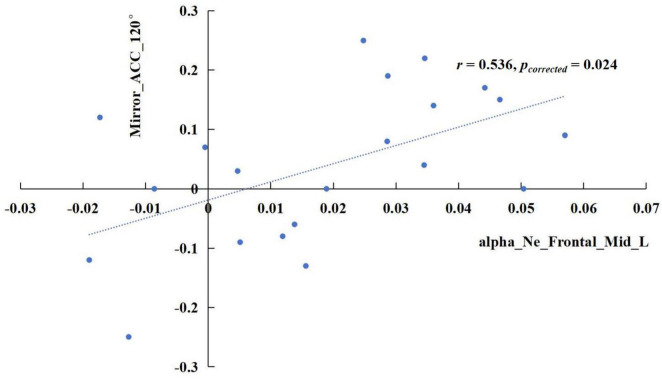
In the alpha band, the difference of nodal efficiency in the left middle frontal gyrus between the BS and TSD state was positively correlated with the difference of ACC in the mirrored 120° state.

**FIGURE 7:**
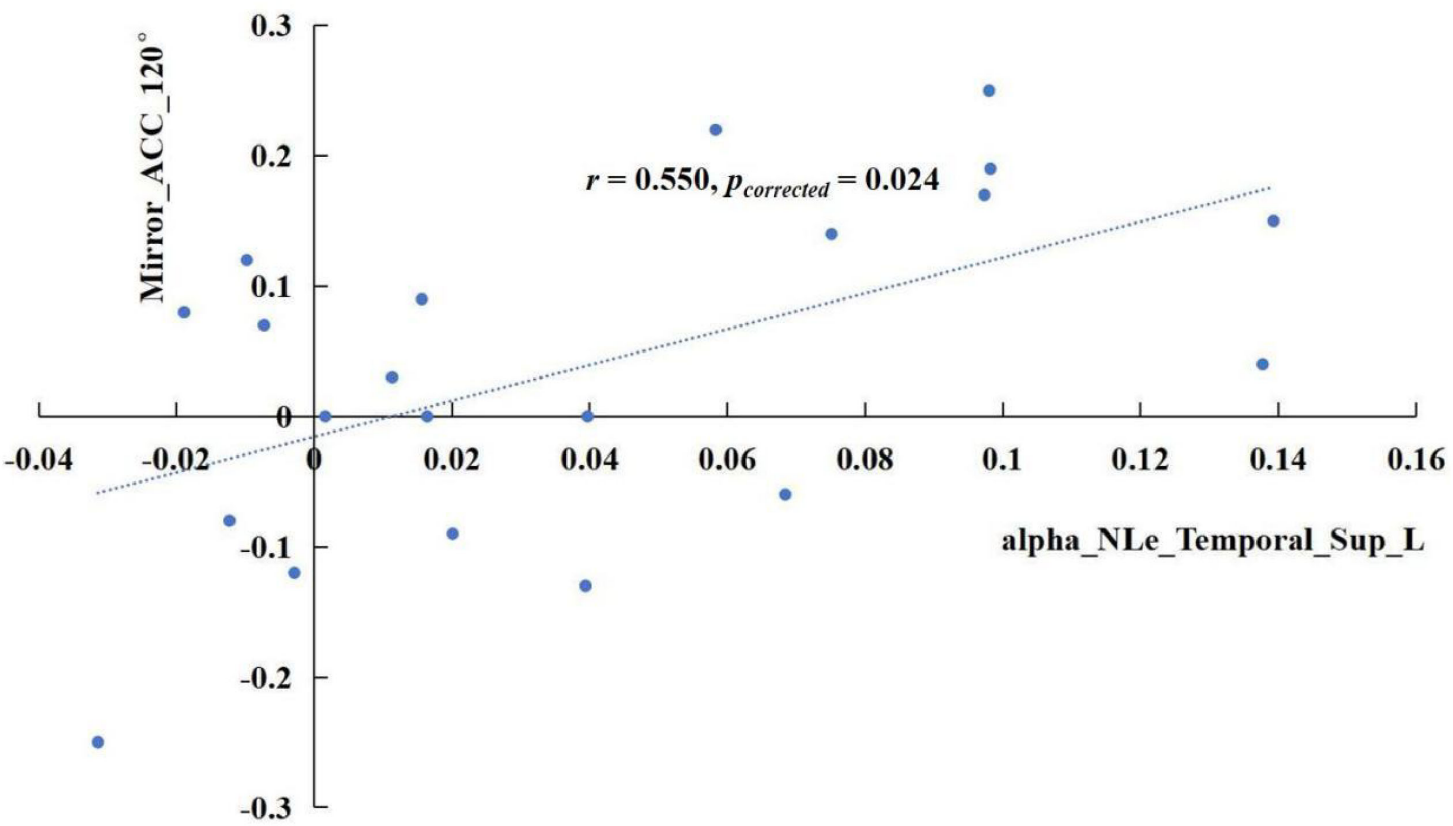
In the alpha band, the difference of nodal local efficiency in the left superior temporal gyrus between the BS and TSD state was positively correlated with the difference of ACC in the mirrored 120° state.

The results revealed significant correlations in specific frequency bands. In the delta band, the increase of sigma values in the TSD state showed a positive correlation with the rise in mirrored 120° ACC (Figure 5), *r* = 0.741, *p*_corrected_ < 0.001. In the alpha band, the enhancement of the nodal efficiency in the left middle frontal gyrus in the TSD state was positively correlated with the advancement of the mirrored 120° ACC (Figure 6), *r* = 0.536, *p*_corrected_ = 0.024. Similarly, the improvement of the nodal local efficiency in the left superior temporal gyrus in the TSD state was positively correlated with the enhancement of mirrored 120° ACC (Figure 7), *r* = 0.550, *p*_corrected_ = 0.024.

## Discussion

The ANOVA results for behavioral data revealed a significant main effect of sleep condition on RT, with significantly longer RT in the TSD condition compared to the BS condition. In the simple effect analysis of the interaction between sleep condition and angle, RT for 60° and 120° images were longer in the TSD condition than in the BS condition. Furthermore, in the simple effect analysis of the three-way interaction, RT for 120° images were significantly longer in the TSD condition compared to the BS condition, regardless of whether the images were normal or mirrored. For ACC, the main effect of sleep condition was not significant. However, in the simple effect analysis of the interaction between sleep condition and image type, the ACC for mirrored images was higher in the TSD condition than in the BS condition. The simple effect analysis of the three-way interaction for ACC revealed that this increase in ACC after TSD was primarily driven by the mirrored 120° images. Compared to the BS condition, RT were significantly longer in the TSD condition, which aligned with our expectation that TSD impaired performance in mental rotation tasks. Individuals needed to continuously focus on the stimulus during the mental rotation task, and TSD significantly reduced the ability to maintain and allocate attention (Lim et al., 2010), which made it difficult for individuals to steadily focus attention on the task for a long time, thus spending more time. However, the ACC for mirrored 120° images significantly increased after TSD. This might be attributed to the higher task difficulty of mirrored images, prompting participants to exert greater effort and adopt a strategy of prolonging RT to maintain higher ACC levels.

The main effect of image type on RT indicated that RT for mirrored images were significantly longer than those for normal images. The simple effect analysis of the interaction between image type and angle showed that RT for mirrored images were longer than those for normal images at 0°, 60°, and 180°. In the three-way interaction analysis, under the BS condition, RT for mirrored images were significantly longer than those for normal images at 0°, 60°, and 180°, while under the TSD condition, this difference was only significant at 60°. For ACC, the main effect of image type was not significant, and no meaningful results were found in the simple effect analysis of the interactions.

The main effect of angle was significant for both RT and ACC. *Post hoc* comparisons revealed the following patterns: RT(120°) > RT(60°) > RT(180°) > RT(0°), ACC(120°) < ACC(60°) < ACC(180°) < ACC(0°). The simple effect analysis of the three-way interactions for both RT and ACC demonstrated that the increasing trend in RT and the decreasing trend in ACC were consistent across all four conditions of 2 (BS, TSD) × 2 (Normal, Mirrored). This indicated that task difficulty decreased progressively from 120° to 60°, 180°, and 0°, consistent with previous research (Bryden et al., 1990).

The statistical analysis of global properties in EEG revealed that the small-world index sigma was significant in the delta, theta, and beta frequency bands, with the sigma values under the TSD condition being significantly higher than that under the BS condition. The clustering coefficient measures the likelihood of connections between a node and its neighboring nodes in the network, while the shortest path length is the average of the minimum number of edges between any two nodes in the network. There are three types of networks in global properties: regular, random, and small-world networks. Regular networks are characterized by high clustering coefficients and long shortest path lengths, which facilitate efficient local information transmission but are inefficient for distant nodes. In contrast, random networks exhibit the opposite characteristics. Small-world networks combine high clustering coefficients with short path lengths, enabling efficient information transmission both locally and globally (Bullmore and Sporns, 2009). The human brain exhibits small-world properties, which are crucial for cognitive functions (Farahani et al., 2019). This study found that small-world properties were enhanced after TSD, consistent with the findings of Liu H. et al. (2014), who observed that enhanced global properties after TSD included not only small-world but also global and local efficiency. This consistency can be explained as a compensatory response of the brain to counteract the cognitive resource deficits following TSD, aiming to maintain normal functionality.

Regarding EEG nodal properties, a widespread decrease was observed across four frequency bands after 36 h of TSD, including regions such as the temporal pole, superior/middle/inferior frontal gyrus in the orbital part, olfactory cortex in the limbic system, and subcortical regions like the amygdala, caudate nucleus, and lenticular nucleus of the putamen. Additionally, as expected, the degree centrality, nodal efficiency, and nodal local efficiency in the dorsal visual pathway showed enhanced responses post-TSD. Specifically, activity differences in the parietal region were mainly concentrated in the delta and theta bands, including the superior parietal lobule (precuneus), inferior parietal lobule (supramarginal and angular gyri), and superior parietal gyrus. In the alpha band, besides the parietal lobe, the nodal efficiency in the middle frontal gyrus and the nodal local efficiency in the superior temporal gyrus were significantly enhanced after TSD. In the beta band, the degree centrality in the cuneus and supramarginal gyrus, as well as the degree centrality and nodal efficiency in the superior occipital gyrus, were also significantly enhanced post-TSD. These results suggest that individuals counteract the excessive fatigue of the visual neural circuits and the overall cognitive resource deficits by enhancing the degree centrality and nodal efficiency in the dorsal visual pathway, particularly in the parietal region, predominantly in the slow-wave frequency bands. Previous EEG studies have found high slow-wave activity in the parieto-occipital regions following TSD (Kurth et al., 2016). A prior fMRI study also noted that while TSD led to increased RT and decreased ACC in the Attention Network Test, the percent amplitude of fluctuation (PerAF) in the bilateral visual and sensorimotor cortices increased post-TSD compared to pre-TSD, with these changes in brain activity showing high discriminative power in distinguishing between BS and TSD states, aligning with our findings (Zeng et al., 2021). However, we did not observe significant compensatory activities in the ventral visual pathway. Previous surface morphological analyses have shown that only the parieto-occipital cortex thins after TSD, and this morphological change is negatively correlated with sleepiness (Liu C. et al., 2014; Long et al., 2020; Wang C. et al., 2021). This may be because the ventral pathway is less sensitive to TSD, and the dorsal pathway overlaps with the frontoparietal attention network, possibly carrying more advanced functions in visual cognition that require greater effort, making the dorsal pathway more susceptible to the effects of TSD.

The correlation results revealed that the enhancement of delta-band sigma after TSD showed a positive correlation with the improvement of ACC in the mirrored 120° trials. Furthermore, increased nodal efficiency in the middle frontal gyrus and enhanced nodal local efficiency in the superior temporal gyrus within the alpha band were both positively associated with improved ACC in the mirrored 120° trials. These findings regarding sigma activity provide additional evidence supporting the view that the brain compensates for TSD-induced impairment in visuospatial cognition by enhancing small-world properties, that is through improved global network efficiency.

Interestingly, our results revealed the compensatory roles of the middle frontal gyrus and superior temporal gyrus in spatial cognition following TSD, which are not typically associated with the classical dorsal-ventral visual pathways. According to the AAL atlas, the middle frontal gyrus is classified within the prefrontal cortex. In mental rotation tasks, prefrontal involvement has been linked to working memory processes (Gogos et al., 2010) and attentional control (Koshino et al., 2005), as individuals must compare angular differences of objects before and after rotation. The enhanced nodal efficiency in the middle frontal gyrus suggests a significant reduction in path length between this region and other nodes following TSD, thereby improving information transfer efficiency. This phenomenon may represent a top-down control mechanism for visual cognitive processing. The superior temporal gyrus plays a crucial role in multisensory information processing. Research has demonstrated functional connectivity between the superior temporal gyrus and the inferior parietal lobule, which is essential for integrating audiovisual information and translating it into motor actions (Sun et al., 2022). The increased nodal local efficiency in the superior temporal gyrus indicates enhanced connection strength and information transfer rate among adjacent brain regions following TSD. Given its anatomical proximity to the supramarginal gyrus, angular gyrus, and middle temporal gyrus, these findings suggest compensatory activities within the dorsal-ventral visual pathways after TSD.

According to the review by Kanishka and Jha (2022), memory can be divided into long-term memory and short-term memory. Short-term memory includes working memory; long-term memory encompasses declarative memory (i.e., explicit cognition of facts and events) and non-declarative memory (unconscious knowledge acquired through procedural learning, priming effects, etc.). Mental rotation involves how to efficiently manipulate the mental image of an object and is an internalized skill. Therefore, the practice of mental rotation tasks is stored in procedural memory, and TSD may impair the consolidation of procedural memory (Spanò et al., 2018). Memory consolidation refers to the process by which memory traces gradually transform from a short-term unstable state to a long-term stable state after encoding is completed, accompanied by dynamic changes in experience-dependent internal representations and their neurobiological bases (Klinzing et al., 2019). Memory consolidation mainly occurs during wakeful rest and sleep (Humiston et al., 2019; Wang S. Y. et al., 2021). A large number of studies have demonstrated that sleep can promote memory consolidation. Slow-wave sleep plays a crucial role in the consolidation of declarative memory (Gais et al., 2006), and rapid eye movement (REM) sleep can facilitate the consolidation of procedural memory (Spanò et al., 2018). However, TSD can impair the memory consolidation process (Yang et al., 2015; Abel et al., 2013).

The consolidation of declarative memory involves the hippocampus and medial temporal lobe structures, while non-declarative memory relies on structures such as the neocortex, amygdala, and cerebellum (Kanishka and Jha, 2022). Research indicates that memory consolidation can still occur through certain compensatory circuits even in the presence of damage to key brain regions. For instance, the dorsal hippocampus (DH) plays a crucial role in the consolidation of contextual fear-conditioned memory. Contextual fear conditioning training involving the DH significantly reduces REM sleep in rats; however, in the absence of DH, rats can still form compensatory contextual fear memory without changes in REM sleep (Kant and Jha, 2019). Further studies (Kant and Jha, 2023) have shown that the compensatory contextual fear memory pathway begins to form 3 days after the conditioning trial, but not after 1 day, and the infralimbic cortex plays a key role in inducing and subsequent development of compensatory contextual fear responses after DH loss. Based on this, it can be speculated that although TSD may impair the consolidation process of procedural memory for mental rotation skills, it may also compensate for it through other pathways. Currently, no scholars have explored this issue. In future research, we can set up control groups and combine electrophysiological and brain imaging techniques to investigate the impact of TSD on memory consolidation.

Furthermore, research indicates that memory consolidation can affect sleep structure. Kumar and Jha (2017) found that after the consolidation of cue-induced fear conditioning memory, non-rapid eye movement (NREM) sleep was significantly prolonged, but when this memory consolidation was disrupted, no similar changes occurred in NREM sleep. Tripathi et al. (2018) discovered that after appetitive conditioning, REM sleep increased significantly and was positively correlated with performance on the appetitive conditioning task. They speculated that the enhanced REM sleep after conditioning might be necessary for consolidating appetitive conditioning memory. This suggests that procedural memory consolidation following a mental rotation task may alter sleep structure. In future studies, we can set up a control group and monitor the changes in sleep structure during restorative sleep in the TSD group and normal sleep in the control group to explore the impact of TSD on memory consolidation and the influence of memory consolidation on sleep structure.

This article has several limitations. Firstly, due to the relatively low spatial resolution of the EEG method, the graph theory analysis based on source localization methods carries certain spatial inaccuracies, and the precision of brain region localization is not as high as that of fMRI methods. Secondly, the brain regions involved in the dorsal and ventral visual pathways are responsible for a wide range of complex cognitive functions, yet our discussion is solely based on the perspective of visual functionality. Lastly, the EEG data analysis included 21 participants, which may introduce effects associated with a small sample size.

## Conclusion

Behavioral results revealed that, compared to the BS state, RT significantly increased under the TSD condition, indicating impaired spatial cognitive abilities due to TSD. Notably, the ACC in the mirrored 120° trials showed a significant increase under TSD, suggesting that participants exerted greater effort in more challenging tasks and adopted a strategy of prolonging RT to ensure ACC. EEG analysis demonstrated a significant increase in the small-world property index, sigma, following TSD. This enhancement in sigma values positively correlated with the increasement of ACC in the mirrored 120° trials, implying that individuals compensate for cognitive impairments post-TSD by augmenting small-world property. Furthermore, nodal properties in the visual dorsal pathway, particularly in the parietal region, were significantly enhanced after TSD, providing additional evidence for compensatory activities in the visual dorsal pathway following TSD. Additionally, nodal properties in the middle frontal gyrus and superior temporal gyrus were significantly enhanced post-TSD, and these enhancements were positively correlated with the increasement of ACC in the mirrored 120° trials. This suggests that after TSD, there is both top-down control from the middle frontal gyrus over the visual pathway and bottom-up information integration from the superior temporal gyrus.

## Data Availability

The data analyzed in this study is subject to the following licenses/restrictions: The dataset contains sensitive information related to human participants and is subject to ethical and legal restrictions. Participants provided consent for their data to be used solely for research purposes related to this study. The dataset is owned by Beijing Sport University, and its use is restricted to the research team involved in this study. Access to the data is limited to authorized personnel, and public release is not permitted without explicit approval from the data owners. Requests for access can be made by contacting the principal investigator. Requests to access these datasets should be directed to budeshao@aliyun.com.

## Supplementary material

Supplementary Figure 1 Simple effect analysis of RT interaction in Sleep condition × Angle. **p* < 0.05, ***p* < 0.01, ****p* < 0.001, the same below.

Supplementary Figure 2 Simple effect analysis of RT interaction in Image type × Angle.

Supplementary Figure 3 Simple effect analysis of ACC interaction in Sleep condition × Image type.

Supplementary Figure 4 Simple effect analysis of ACC interaction in Image type × Angle.
