# Supplementary material for: The enhancement of nodal properties in the dorsal visual pathway is associated with compensatory mechanisms of visuospatial cognitive abilities following total sleep deprivation
Source: Front Neurosci. 2025 Sep 4;19:1585763. doi: 10.3389/fnins.2025.1585763 (PMC12443831; doi:10.3389/fnins.2025.1585763)
Supplement: Supplementary file 1 [file Table_1.docx]

Supplementary Table 1. Paired t test statistical results of nodal properties in the delta band.

| brain regions | p(Dc) | t(Dc) | p(Ne) | t(Ne) | p(NCp) | t(NCp) | p(NLe) | t(NLe) |
| --- | --- | --- | --- | --- | --- | --- | --- | --- |
| Frontal_Sup_Orb_L | —— | —— | —— | —— | <0.001 | 4.31 | <0.001 | 3.862 |
| Frontal_Sup_Orb_R | 0.003 | 3.339 | —— | —— | —— | —— | —— | —— |
| Frontal_Mid_Orb_L | —— | —— | —— | —— | <0.001 | 4.666 | <0.001 | 3.974 |
| Olfactory_L | <0.001 | 4.046 | 0.001 | 3.833 | —— | —— | —— | —— |
| Olfactory_R | <0.001 | 6.531 | <0.001 | 6.385 | —— | —— | —— | —— |
| Frontal_Med_Orb_L | —— | —— | —— | —— | <0.001 | 4.738 | <0.001 | 4.345 |
| Frontal_Med_Orb_R | 0.004 | 3.263 | 0.003 | 3.317 | —— | —— | —— | —— |
| Rectus_L | 0.007 | 2.980 | —— | —— | <0.001 | 4.305 | <0.001 | 4.201 |
| Rectus_R | 0.002 | 3.647 | —— | —— | —— | —— | —— | —— |
| Insula_R | 0.008 | 2.955 | 0.003 | 3.38 | —— | —— | —— | —— |
| Amygdala_R | <0.001 | 4.661 | <0.001 | 5.136 | —— | —— | —— | —— |
| SupraMarginal_L | <0.001 | -4.245 | <0.001 | -4.094 | —— | —— | —— | —— |
| Angular_L | 0.005 | -3.123 | —— | —— | —— | —— | —— | —— |
| Caudate_R | <0.001 | 4.166 | 0.001 | 3.796 | —— | —— | —— | —— |
| Putamen_R | 0.001 | 3.734 | <0.001 | 3.869 | —— | —— | —— | —— |
| Pallidum_R | <0.001 | 4.399 | <0.001 | 4.406 | —— | —— | —— | —— |
| Temporal_Pole_Sup_R | 0.001 | 3.805 | <0.001 | 4.133 | —— | —— | —— | —— |
| Temporal_Pole_Mid_R | <0.001 | 4.095 | <0.001 | 4.390 | —— | —— | —— | —— |

Supplementary Table 2. Paired t test statistical results of nodal properties in the theta band.

| brain regions | p(Dc) | t(Dc) | p(Ne) | t(Ne) | p(NCp) | t(NCp) | p(NLe) | t(NLe) |
| --- | --- | --- | --- | --- | --- | --- | --- | --- |
| Frontal_Inf_Oper_R | —— | —— | —— | —— | —— | —— | 0.003 | 3.352 |
| Frontal_Inf_Orb_R | 0.002 | 3.579 | 0.001 | 3.753 | —— | —— | 0.004 | 3.206 |
| Olfactory_L | <0.001 | 3.906 | 0.001 | 3.840 | —— | —— | —— | —— |
| Olfactory_R | <0.001 | 4.936 | <0.001 | 4.672 | —— | —— | —— | —— |
| Frontal_Med_Orb_L | —— | —— | —— | —— | 0.002 | 3.598 | 0.000 | 4.596 |
| Frontal_Med_Orb_R | 0.004 | 3.231 | 0.003 | 3.359 | —— | —— | —— | —— |
| Rectus_R | 0.001 | 3.707 | 0.006 | 3.089 | —— | —— | —— | —— |
| Insula_R | 0.001 | 3.745 | 0.001 | 3.720 | —— | —— | —— | —— |
| Cingulum_Ant_L | 0.005 | 3.171 | 0.007 | 2.978 | —— | —— | —— | —— |
| Amygdala_R | <0.001 | 4.430 | 0.000 | 4.518 | —— | —— | —— | —— |
| Parietal_Sup_L | —— | —— | 0.008 | -2.961 | —— | —— | —— | —— |
| SupraMarginal_L | —— | —— | 0.006 | -3.085 | —— | —— | —— | —— |
| Angular_L | —— | —— | 0.009 | -2.911 | —— | —— | —— | —— |
| Precuneus_L | 0.004 | -3.228 | 0.008 | -2.944 | —— | —— | —— | —— |
| Caudate_L | —— | —— | —— | —— | <0.001 | 4.406 | 0.000 | 4.531 |
| Caudate_R | 0.003 | 3.333 | 0.006 | 3.059 | —— | —— | 0.001 | 3.702 |
| Putamen_L | —— | —— | —— | —— | 0.001 | 3.975 | 0.000 | 4.760 |
| Putamen_R | 0.003 | 3.426 | 0.004 | 3.226 | —— | —— | —— | —— |
| Pallidum_L | —— | —— | —— | —— | 0.001 | 3.851 | 0.000 | 5.116 |
| Pallidum_R | 0.002 | 3.611 | 0.002 | 3.516 | —— | —— | —— | —— |
| Thalamus_R | —— | —— | —— | —— | —— | —— | 0.003 | 3.317 |
| Temporal_Pole_Sup_R | <0.001 | 4.519 | <0.001 | 4.490 | —— | —— | —— | —— |
| Temporal_Pole_Mid_R | <0.001 | 5.432 | <0.001 | 5.452 | —— | —— | —— | —— |

Supplementary Table 3. Paired t test statistical results of nodal properties in the alpha band.

| brain regions | p(Dc) | t(Dc) | p(Ne) | t(Ne) | p(NCp) | t(NCp) | p(NLe) | t(NLe) |
| --- | --- | --- | --- | --- | --- | --- | --- | --- |
| Frontal_Sup_Orb_L | —— | —— | —— | —— | —— | —— | 0.006 | 3.110 |
| Frontal_Sup_Orb_R | <0.001 | 5.022 | <0.001 | 4.921 | —— | —— | 0.003 | 3.443 |
| Frontal_Mid_L | —— | —— | 0.002 | -3.649 | —— | —— | —— | —— |
| Frontal_Mid_Orb_L | —— | —— | —— | —— | —— | —— | 0.001 | 4.071 |
| Frontal_Mid_Orb_R | —— | —— | —— | —— | —— | —— | 0.002 | 3.566 |
| Frontal_Inf_Orb_R | 0.001 | 3.872 | —— | —— | —— | —— | —— | —— |
| Olfactory_L | 0.001 | 4.117 | 0.003 | 3.468 | —— | —— | 0.002 | 3.505 |
| Frontal_Med_Orb_L | —— | —— | —— | —— | —— | —— | 0.002 | 3.695 |
| Frontal_Med_Orb_R | 0.001 | 4.065 | 0.002 | 3.512 | —— | —— | 0.001 | 3.769 |
| Rectus_L | <0.001 | 5.309 | <0.001 | 4.455 | —— | —— | 0.002 | 3.487 |
| Rectus_R | <0.001 | 5.071 | 0.001 | 4.071 | —— | —— | 0.006 | 3.120 |
| Amygdala_L | <0.001 | 5.162 | 0.002 | 3.664 | —— | —— | —— | —— |
| SupraMarginal_L | —— | —— | 0.001 | -4.080 | —— | —— | —— | —— |
| Precuneus_L | —— | —— | 0.005 | -3.187 | —— | —— | —— | —— |
| Putamen_L | —— | —— | —— | —— | —— | —— | 0.002 | 3.694 |
| Pallidum_L | 0.002 | 3.691 | —— | —— | —— | —— | —— | —— |
| Thalamus_R | —— | —— | —— | —— | 0.000 | 4.439 | <0.001 | 4.701 |
| Temporal_Sup_L | —— | —— | —— | —— | —— | —— | 0.003 | -3.345 |
| Temporal_Pole_Mid_R | 0.003 | 3.436 | —— | —— | —— | —— | —— | —— |

Supplementary Table 4. Paired t test statistical results of nodal properties in the beta band.

| brain regions | p(Dc) | t(Dc) | p(Ne) | t(Ne) | p(NCp) | t(NCp) | p(NLe) | t(NLe) |
| --- | --- | --- | --- | --- | --- | --- | --- | --- |
| Frontal_Sup_Orb_R | 0.007 | 3.048 | —— | —— | —— | —— | —— | —— |
| Frontal_Mid_Orb_R | 0.003 | 3.440 | 0.002 | 3.646 | —— | —— | —— | —— |
| Frontal_Inf_Orb_R | 0.002 | 3.682 | —— | —— | —— | —— | —— | —— |
| Rolandic_Oper_R | —— | —— | —— | —— | 0.002 | 3.705 | 0.001 | 4.066 |
| Olfactory_L | 0.001 | 4.096 | 0.001 | 3.799 | —— | —— | 0.001 | 3.795 |
| Olfactory_R | <0.001 | 4.675 | <0.001 | 4.297 | —— | —— | —— | —— |
| Rectus_L | 0.003 | 3.405 | 0.005 | 3.214 | —— | —— | —— | —— |
| Rectus_R | 0.001 | 3.757 | 0.005 | 3.233 | —— | —— | —— | —— |
| Amygdala_L | —— | —— | —— | —— | —— | —— | 0.001 | 3.932 |
| Amygdala_R | 0.002 | 3.722 | 0.003 | 3.414 | —— | —— | —— | —— |
| Cuneus_R | 0.004 | -3.303 | —— | —— | —— | —— | —— | —— |
| Occipital_Sup_R | 0.005 | -3.237 | 0.004 | -3.292 | —— | —— | —— | —— |
| SupraMarginal_L | 0.002 | -3.523 | —— | —— | —— | —— | —— | —— |
| Caudate_L | —— | —— | —— | —— | <0.001 | 4.711 | <0.001 | 5.141 |
| Putamen_L | —— | —— | —— | —— | <0.001 | 4.312 | <0.001 | 4.891 |
| Pallidum_L | —— | —— | —— | —— | <0.001 | 4.296 | <0.001 | 4.716 |
| Pallidum_R | 0.007 | 3.020 | —— | —— | —— | —— | —— | —— |
| Heschl_R | —— | —— | —— | —— | —— | —— | 0.004 | 3.357 |
| Temporal_Pole_Sup_R | <0.001 | 4.321 | <0.001 | 4.281 | —— | —— | —— | —— |
| Temporal_Pole_Mid_R | 0.001 | 4.167 | 0.001 | 4.096 | —— | —— | —— | —— |
