# Supplementary figures and images for: The enhancement of nodal properties in the dorsal visual pathway is associated with compensatory mechanisms of visuospatial cognitive abilities following total sleep deprivation
Source: Front Neurosci. 2025 Sep 4;19:1585763. doi: 10.3389/fnins.2025.1585763 (PMC12443831; doi:10.3389/fnins.2025.1585763)

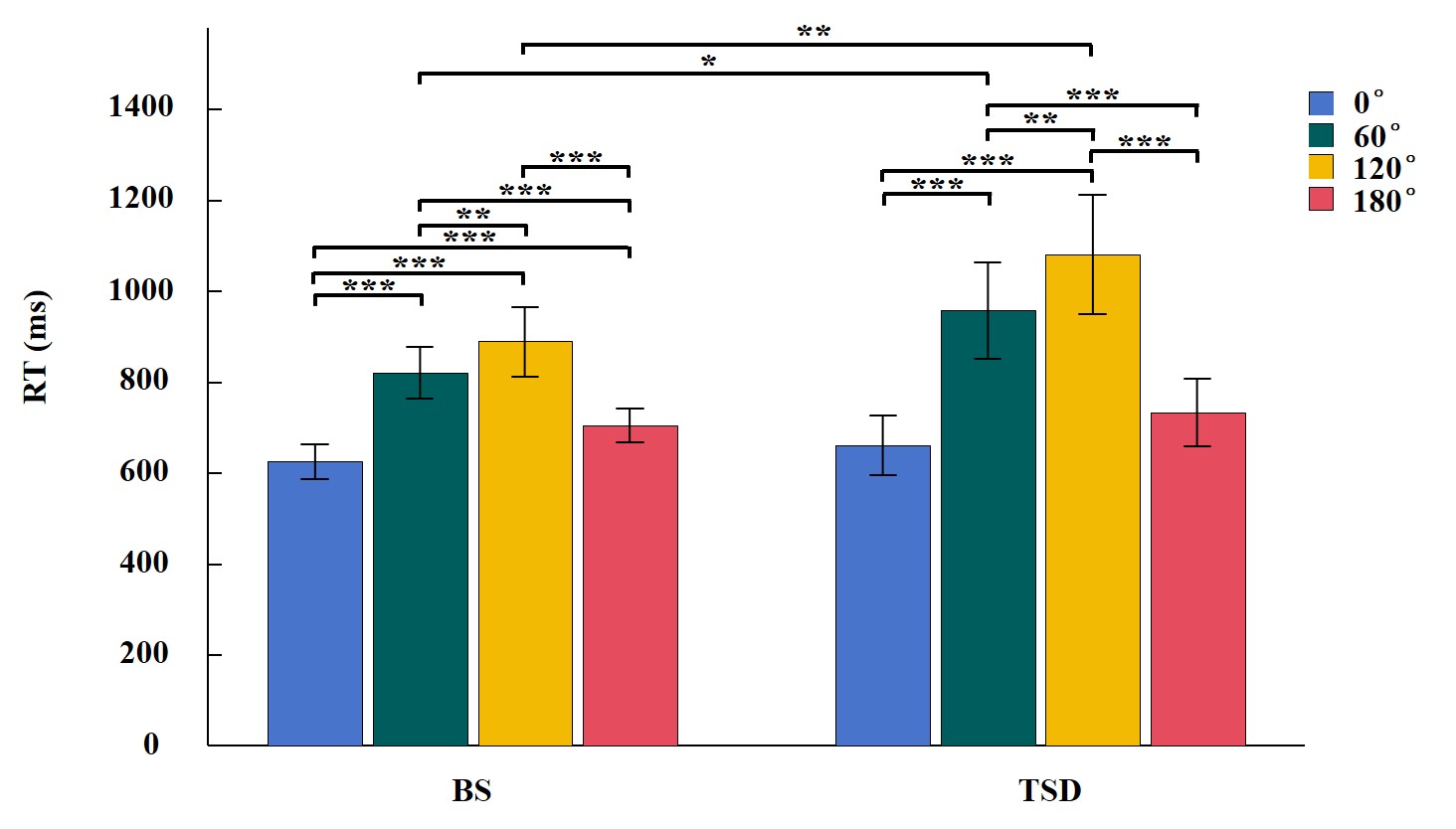

Supplement: Supplementary Figure 1 — Simple effect analysis of RT interaction in Sleep condition × Angle. *p < 0.05, **p < 0.01, ***p < 0.001, the same below. [file Image_1.jpeg]

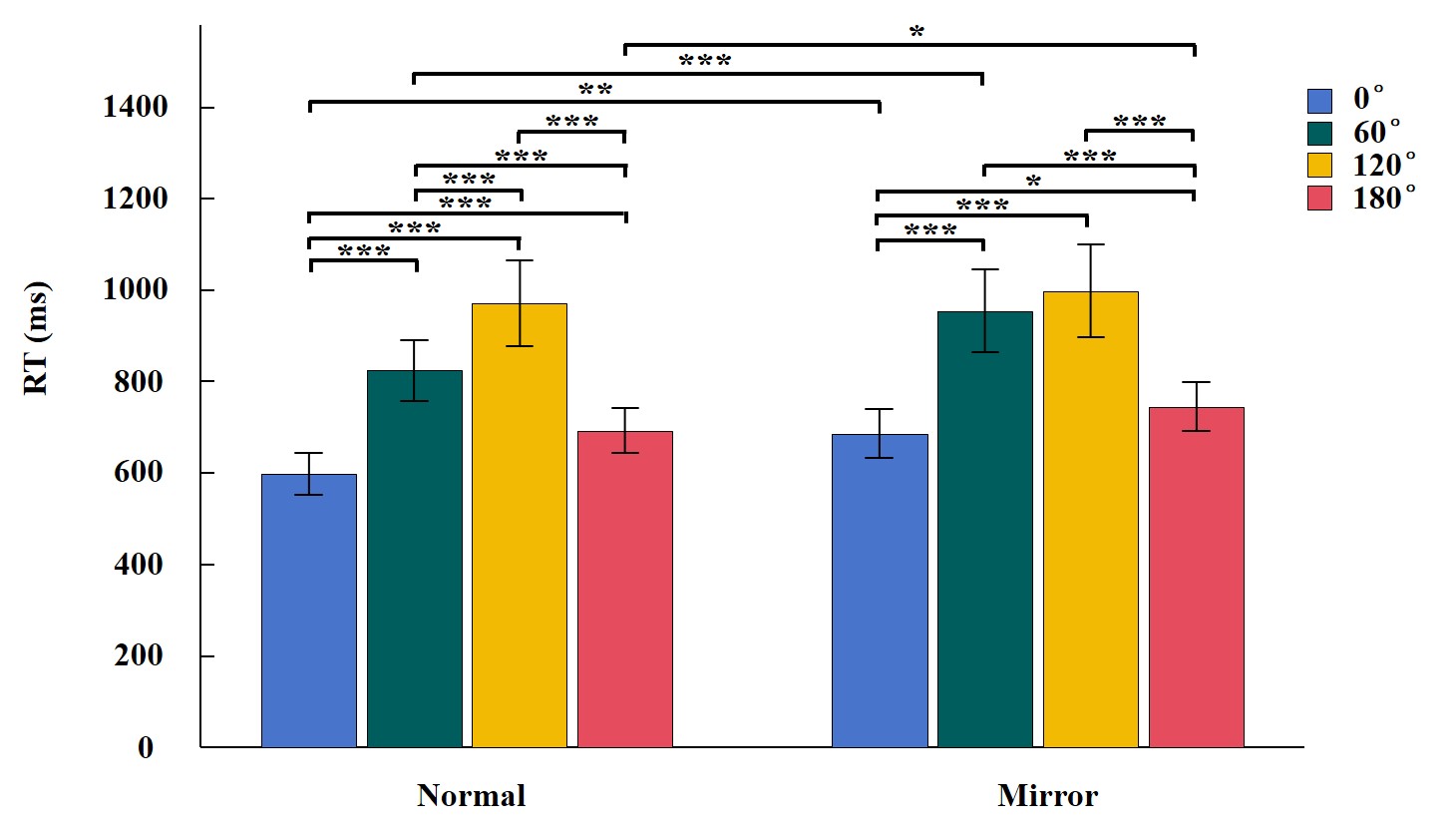

Supplement: Supplementary Figure 2 — Simple effect analysis of RT interaction in Image type × Angle. [file Image_2.jpeg]

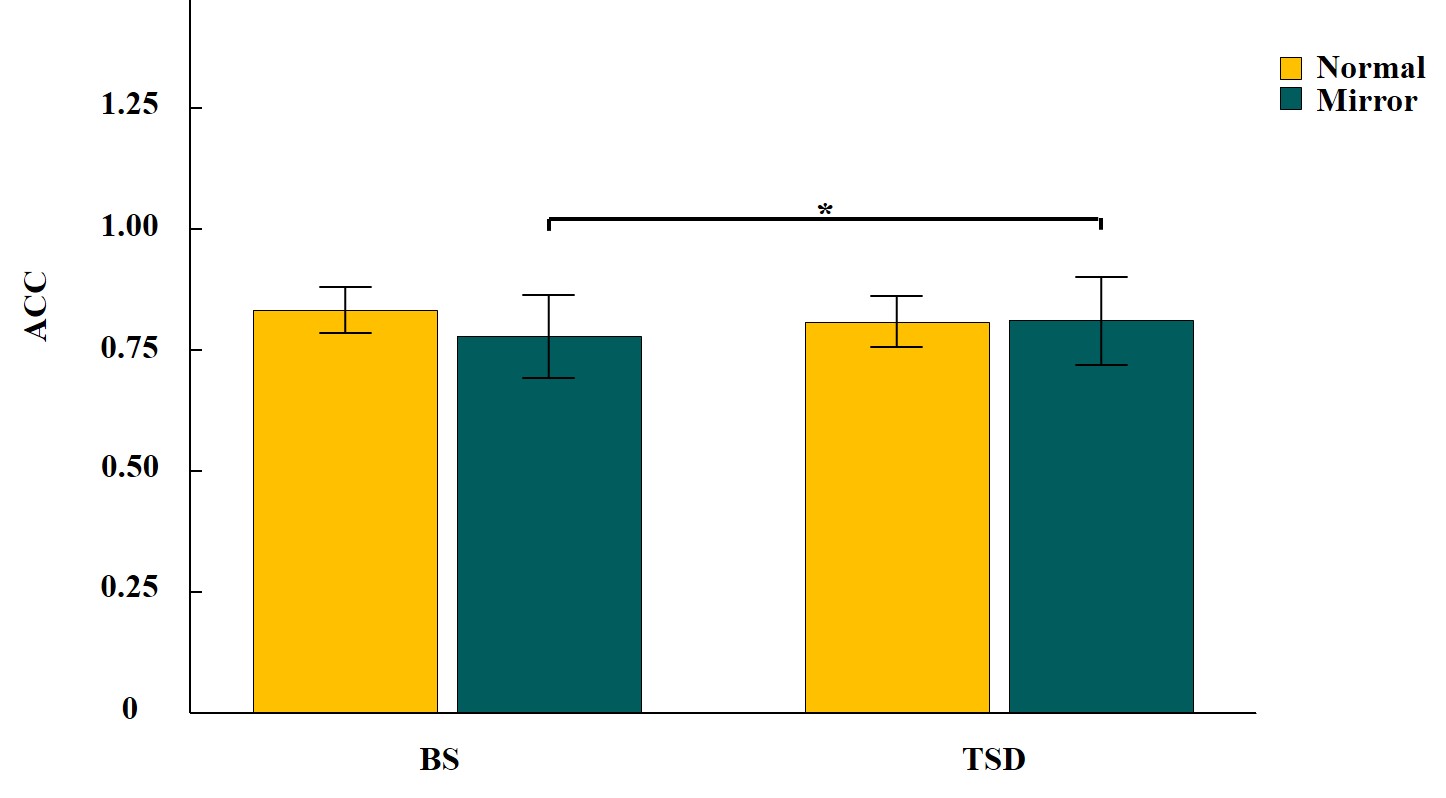

Supplement: Supplementary Figure 3 — Simple effect analysis of ACC interaction in Sleep condition × Image type. [file Image_3.jpeg]

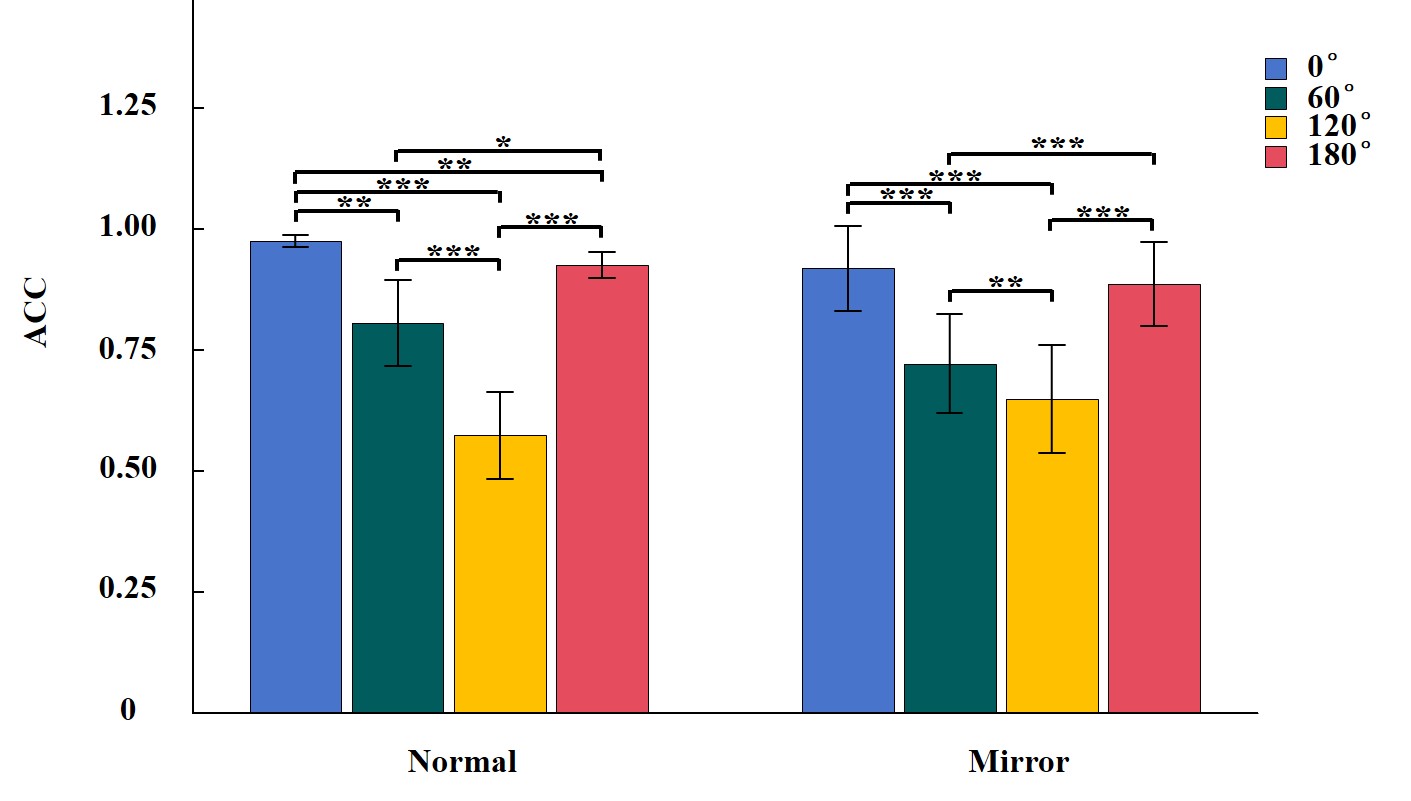

Supplement: Supplementary Figure 4 — Simple effect analysis of ACC interaction in Image type × Angle. [file Image_4.jpeg]
